# Design of a Planar Sensor Based on Split-Ring Resonators for Non-Invasive Permittivity Measurement

**DOI:** 10.3390/s23115306

**Published:** 2023-06-02

**Authors:** Mohammad Alibakhshikenari, Bal S. Virdee, Taha A. Elwi, Innocent D. Lubangakene, Renu K. R. Jayanthi, Amer Abbood Al-Behadili, Zaid A. Abdul Hassain, Syed Mansoor Ali, Giovanni Pau, Patrizia Livreri, Sonia Aïssa

**Affiliations:** 1 Department of Signal Theory and Communications, Universidad Carlos III de Madrid, 28911 Leganés, Madrid, Spain; 2 School of Computing and Digital Media, Center for Communications Technology, London Metropolitan University, 166-220 Holloway Road, London N7 8DB, UKidl0020@my.londonmet.ac.uk (I.D.L.); rer0266@my.londonmet.ac.uk (R.K.R.J.); 3 Communication Engineering Department, Al-Ma’ Moon University College, Baghdad 1004, Iraq; tahaelwi82@almamonuc.edu.iq; 4 International Applied and Theoretical Research Centre (IATRC), Baghdad 10001, Iraq; 5 Electrical Engineering, College of Engineering, Mustansiriyah University, Baghdad 10052, Iraq; 6 Department of Physics and Astronomy, College of Science, King Saud University, P.O. Box 2455, Riyadh 11451, Saudi Arabia; 7 Faculty of Engineering and Architecture, Kore University of Enna, 94100 Enna, Italy; 8 Department of Engineering, University of Palermo, viale delle Scienze BLDG 9, 90127 Palermo, Italy; 9 Institut National de la Recherche Scientifique (INRS), University of Québec, Montréal, QC H5A 1K6, Canada

**Keywords:** split-ring resonator, sensor, complex permittivity, microstrip technology

## Abstract

The permittivity of a material is an important parameter to characterize the degree of polarization of a material and identify components and impurities. This paper presents a non-invasive measurement technique to characterize materials in terms of their permittivity based on a modified metamaterial unit-cell sensor. The sensor consists of a complementary split-ring resonator (C-SRR), but its fringe electric field is contained with a conductive shield to intensify the normal component of the electric field. It is shown that by tightly electromagnetically coupling opposite sides of the unit-cell sensor to the input/output microstrip feedlines, two distinct resonant modes are excited. Perturbation of the fundamental mode is exploited here for determining the permittivity of materials. The sensitivity of the modified metamaterial unit-cell sensor is enhanced four-fold by using it to construct a tri-composite split-ring resonator (TC-SRR). The measured results confirm that the proposed technique provides an accurate and inexpensive solution to determine the permittivity of materials.

## 1. Introduction

Understanding of the dielectric properties of materials is very important for applications in various sectors including food processing industries, agriculture, bio-medical applications, and chemical and defense industries [1,2,3]. A material can be characterized by its complex permittivity, which indicates the extent to which the material can be polarized by an electric field. Compared to low-frequency bands, the microwave band can be used to realize electric fields with much smaller resonance circuits, and a microwave signal is a nonionizing radiation. Although at low power the penetration of microwaves into materials is limited, this is sufficient for the waves to interact with the material to characterize it in a non-invasive modality. The key parameter that is commonly used to determine the permittivity of a material is the shift in the frequency of the resonance circuits or sensor. Recently, planar microwave resonators have increasingly been investigated to develop sensors for real-time characterization of materials and to determine the composition of different mixtures such as ethanol and methanol [4,5].

Popular types of planar sensors are based on dual split-ring resonators (SRR) and complementary split-ring resonators (C-SRR) [6,7,8,9]. This is because these resonators are low profile, non-destructive, can be realized on a planar dielectric medium, and can interface easily between each other and with the circuit of lumped elements. Moreover, these resonators create a high-intensity electric and magnetic field which is necessary to characterize a sample in terms of its permittivity and permeability.

The present work is an extension of our previous work in [10]. The proposed unit-cell sensor, which is based on the complementary split-ring resonator (C-SRR), is modified to concentrate the fringe electric field. The sensor is shown to exhibit metamaterial properties of negative permittivity (*ε_r_*) and negative permeability (*μ_r_*). The sensor’s sensitivity is significantly enhanced by using it to realize a tri-composite split-ring resonator (TC-SRR). It is shown that TC-SRR has a Q-factor that is quadruple that of [10], which is important for accurate permittivity measurements. It is also shown that orthogonal orientation of the consecutive unit-cells in the TC-SRR structure causes its fundamental mode to split with a better Q-factor, but this is at the cost of a higher transmission loss. The TC-SRR was analyzed using a 3D full-wave electromagnetic solver based on the method of moments technique by CST Microwave Studio. The performance and accuracy of the TC-SRR was validated through measurements against known materials.

## 2. Complementary Split-Ring Resonator (SRR)

**Figure 1:**
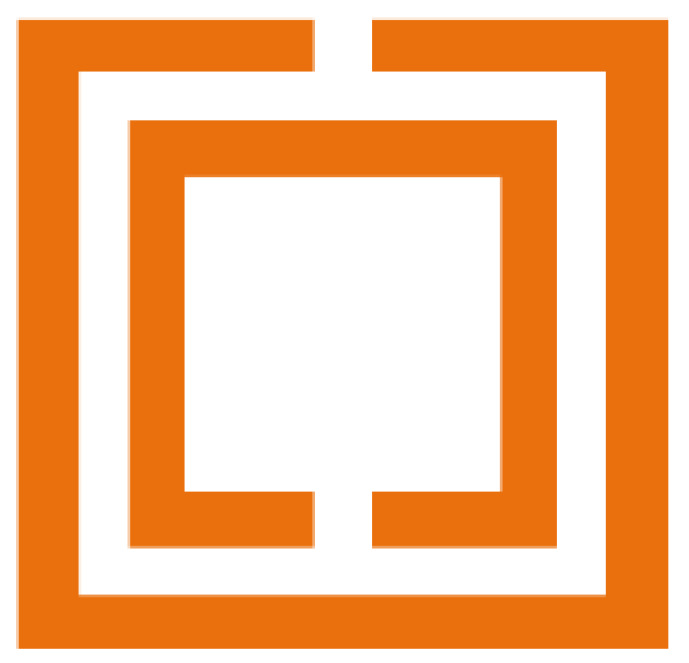
Geometrical structure of a split-ring resonator (SRR) unit-cell.

The properties of artificial materials referred to as metamaterials were first described by V. Veselago in 1967 [11]. He theoretically showed that metamaterials exhibit negative permittivity and negative permeability to electromagnetic waves. These properties cause electromagnetic waves on the material to propagate backwards. Pendry, in 1999, proposed a technique to create negative permeability based on conductive double split-ring resonators (SRR), shown in Figure 1 [12]. The double SRR structure consists of concentric split-ring resonators where the smaller ring nests inside the larger ring. The two resonators are electromagnetically coupled to each other. The equivalent circuit model of the single ring configuration is that of the *RLC* resonator with resonant frequency *ω_o_* = 1/√(*LC*) [13]. The double SRR is essentially equivalent to the single SRR if mutual coupling is weak, because the dimensions of the two rings are very close to each other, resulting in a combined resonance frequency close to that of the single SRR, with the same dimensions but with a larger magnetic moment due to higher current density. The size of the SRR is independent of its wavelength (0.1*λ*). Hence, compared to *λ*/2 transmission line resonators, the SRR is significantly smaller.

The dielectric properties of materials can be determined from the resonance technique [14,15,16,17,18,19,20,21,22,23,24]. This is possible because the resonance frequencies of a resonator are uniquely determined by its geometry, material properties (dielectric substrate on which the resonator is fabricated), and boundary conditions. If these parameters are known, it is then possible to extract the material properties such as permittivity of an unknown material or substance. This technique can be exploited only if the electromagnetic fields of the resonance structure protrude outside the resonator. This is the case of SRR based on microstrip technology. The resonance frequency and insertion-loss of the resonator are perturbed by placing the sample material on top of the resonator. The degree of parameter perturbation will be determined by the dielectric property of the sample material. The resonator can be calibrated with known materials and these data can be used to ascertain the material properties of the unknown sample material. The main advantage of using the proposed TC-SRR is that it magnifies the normal component of the electrical field, which makes it highly responsive to the dielectric part of the material under test (MUT). Consequently, the dielectric properties can be measured more accurately.

## 3. Theoretical Analysis of a Split-Ring Resonator Unit-Cell

**Figure 2:**
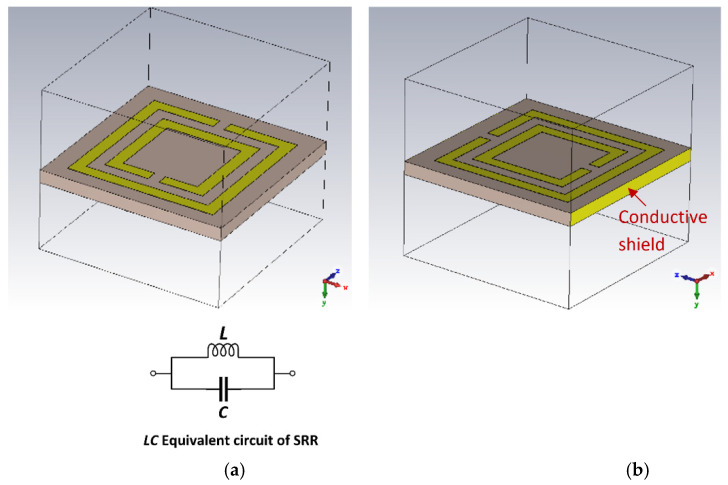
(**a**) Conventional complementary (C-SRR) unit-cell and its simplified equivalent circuit model, and (**b**) modified unit-cell (M-SRR) with field containment shield.

The conventional complementary SRR and its simplified equivalent circuit model is shown in Figure 2a. The SRR unit-cell has a total inductance (*L*) and capacitance (*C*). The total impedance (*Z_T_*) and the resonant frequency (*f_r_*) are given by:

(1) $$Z_{T}=\frac{j\omega L}{1-\omega^{2}LC}$$

(2) $$f_{r}=\frac{1}{2\pi \sqrt{LC}}$$

**Figure 3:**
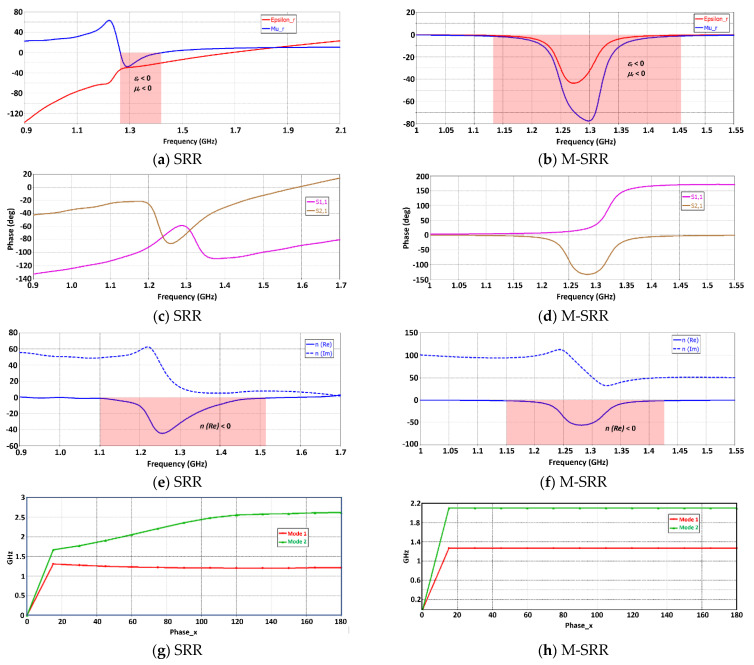
Characteristics of the conventional complementary split-ring resonator (SRR) and modified SRR unit-cell (M-SRR).

The proposed SRR unit-cell, shown in Figure 2b, has a conductive shield on non-periodic sides to prevent the leakage of the electric field. The characteristics of the SRR unit-cell and the modified SRR unit-cell (M-SRR) are shown in Figure 3. The SRR unit-cell exhibits a negative permittivity and permeability between 1.26 GHz and 1.42 GHz, and the M-SRR has negative permittivity and permeability between 1.13 GHz and 1.46 GHz. As evident in Figure 3a,b, the bandwidth of the M-SRR is more than twice the conventional complementary SRR. The S-parameters of the SRR in Figure 3c show the direction of phase change in the transmission coefficient (S_21_), which occurs between 1.18 GHz and 1.3 GHz. In the case of the M-SRR, the direction of phase change in S_21_ occurs between 1.15 GHz and 1.28 GHz. The refractive index (*n*) of the SRR is negative between 1.1 GHz and 1.51 GHz, and in the case of the M-SRR the refractive index is negative between 1.15 GHz and 1.43 GHz. Figure 3g,h shows how the phase of the first two modes changes with frequency with periodicity in the *x*-direction for the SRR unit-cell and the M-SRR unit-cell. For the complementary C-SRR, mode 2 is dominant at frequencies above 1.3 GHz up to 2.6 GHz, and the phase varies in a non-linear fashion. However, in the case of the M-SRR, mode 2 is dominant above 1.25 GHz and up to 2.1 GHz. Furthermore, above a phase of 15 degrees, the phase remains static for both modes. It is evident from these results that the modified SRR extends the metamaterial properties of negative permittivity and permeability by a factor of two.

**Figure 4:**
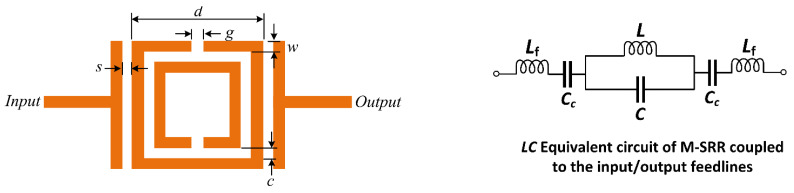
Unit-cell of M-SRR and its simplified equivalent circuit model.

The modified SRR is coupled with the input/output ports with a T-shaped feedline, as shown in Figure 4. In Figure 4, the equivalent circuit of the proposed M-SRR unit-cell is represented as a parallel *LC* circuit which is connected in series with an *L_f_C_c_* circuit that represents the coupled input/output feedlines. The total impedance (*Z_T_*_1_) of this structure is given by:

(3) $$Z_{T,1}=j\frac{\left[2\left(\omega^{2}L_{f}C_{c}-1\right)\left(1-\omega^{2}LC\right)+\omega L\right]}{\omega C_{c}\left(1-\omega^{2}LC\right)}$$

This structure excites two resonance frequencies given by:

(4) $$f_{1}=\frac{1}{2\pi \sqrt{LC}}$$

(5) $$f_{2}=\frac{1}{2\pi \sqrt{L_{f}C_{c}}}$$

**Figure 5:**
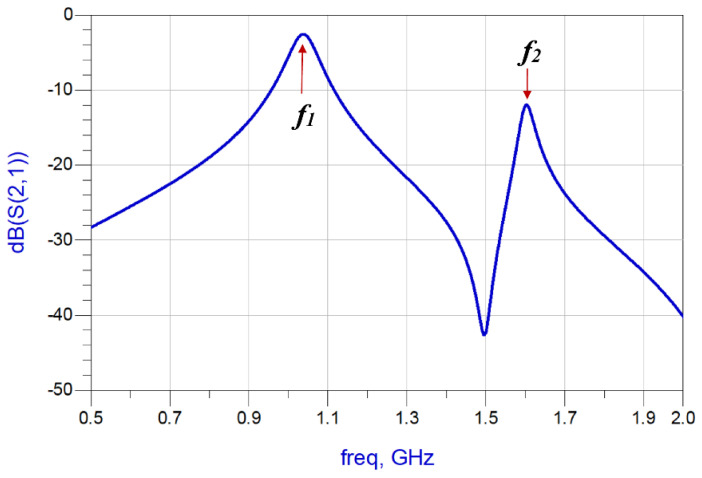
Transmission response of the proposed M-SRR unit-cell.

The modified SRR unit-cell was constructed on FR4-epoxy substrate with *ε_r_* of 4.4 and thickness of 1.6 mm. The thickness of the conductor is 35 microns. The parameters defining the M-SRR in Figure 4 are listed in Table 1. The structure was simulated using 3D full-wave EM solver based on the method of moments technique in CST Microwave Studio. Figure 5 shows the transmission response of the M-SRR. As predicted by the equivalent circuit model, it resonates at two distinct modes at frequencies *f*_1_ and *f*_2_. The lumped elements of the equivalent circuit can be extracted using established techniques.

### 3.1. Sensitivity Analysis of the Unit-Cell M-SRR Sensor

**Figure 6:**
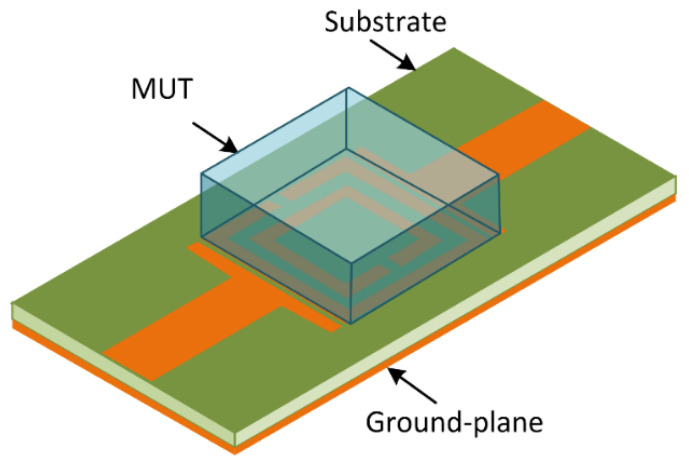
Setup of unit-cell M-SRR sensor with material under test (MUT) placed on it.

Sensitivity analysis was carried out for the proposed M-SRR sensor. This involved locating the sample of the material under test (MUT) of a finite dimension, permittivity and loss tangent on top of the unit-cell sensor, as shown in Figure 6. The sample does not cover the input/output feedlines but only the unit-cell sensor to effectively perturb its E-field. The resonant frequencies of the sensor will vary when the MUT is placed on the sensor. This is because the sensor’s E-field penetrates the MUT. The variations of the resonant frequencies can be expressed in terms of the effective permittivities, as given by:

(6) $$f_{r,MUT}=f_{r,AIR}\sqrt{\frac{\varepsilon_{eff,AIR}}{\varepsilon_{eff,MUT}}}$$

where $f_{r,AIR}$ and $\varepsilon_{eff,AIR}$ are, respectively, the resonant frequency and effective permittivity without MUT, and $f_{r,MUT}$ and $\varepsilon_{eff,MUT}$ are the resonant frequency and effective permittivity when a test material is placed on the sensor, respectively. Through an EM simulation, the resonant frequencies are extracted for different permittivities of MUT.

**Figure 7:**
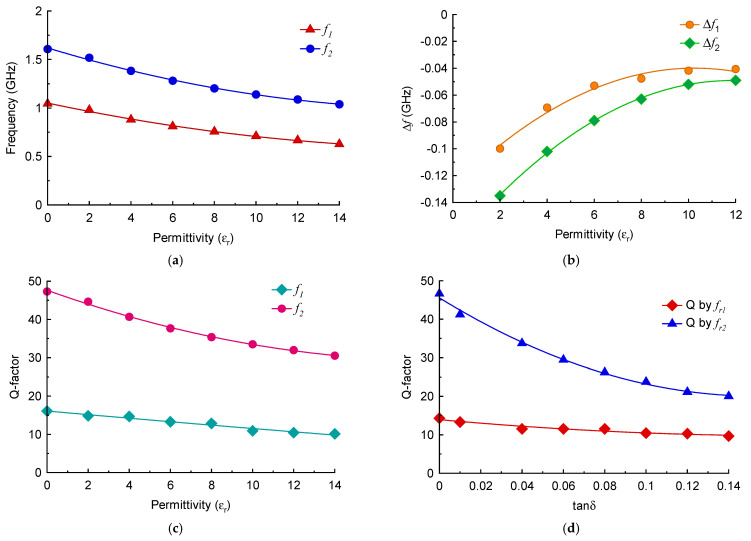
(**a**) Resonant frequency of the M-SRR sensor as a function of MUT permittivity. (**b**) Change in the resonant frequency of the M-SRR sensor as a function of MUT permittivity. (**c**) Q-factor of the two frequency responses with increase in MUT permittivity. (**d**) Q-factor as a function of loss tangent of the MUT.

The variation of the two resonant modes excited in the M-SRR sensor as a function of different permittivity (real part) magnitudes of the MUT is shown in Figure 7a. The height of the MUT samples was fixed at 3 mm. The resonance frequency of both modes declines approximately linearly with the increase in the permittivity value of the MUT. Figure 7b shows that the changes in the resonant frequency of both modes almost converge with increases in the MUT permittivity.

The quality (Q) factor of a resonator Is given by $Q=f_{r}/\Delta f$, where *f_r_* is the resonant frequency, and ∆*f* is the 3 dB bandwidth. The Q-factor of the first resonance mode is significantly lower than the second mode, as is evident in Figure 7c. Compared to the second mode, the Q-factor of the first mode declines marginally in a linear fashion with an increase in MUT permittivity. Figure 7d shows how the Q-factor of both modes is affected by increasing the loss-tangent (tan δ) of the MUT. There is a negligible effect on the Q-factor of the first mode; however, the Q-factor of the second resonance deteriorates rapidly with increase in the loss-tangent. This indicates that the second mode is particularly sensitive to dielectric loss of the MUT.

**Figure 8:**
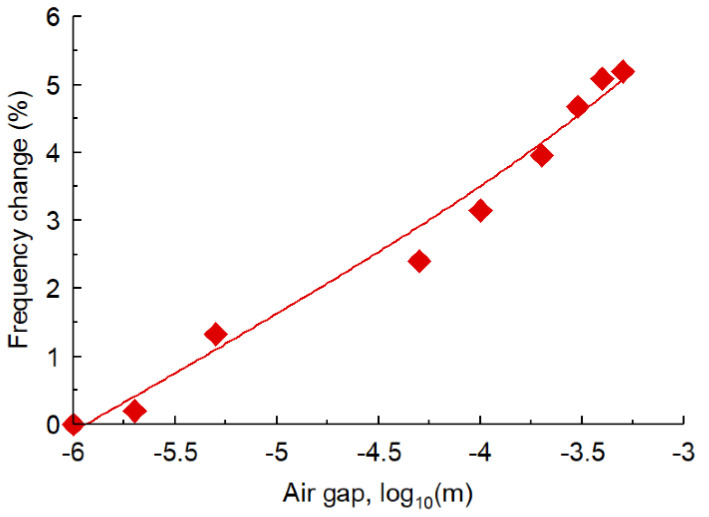
Frequency error of the first resonant mode as a function of air gap between the sensor and the MUT with *ε_r_* = 2 and tan δ = 0.014.

Since the sensor is flat and the sample is a rectangular block with a flat bottom, there is a negligible possibility of having an air gap between them which would otherwise underestimate the permittivity of the MUT. The presence of an air gap will alter the resonator’s load, which will consequently introduce errors in the permittivity measurement. To determine the degree of error introduced by an air gap, it was necessary to conduct a study. Figure 8 shows the percentage change in the resonance frequency of the first mode by the air gap between the sensor and the MUT sample. The results are for an MUT sample with a permittivity of 2 and tan δ of 0.014. The percentage error in the resonant frequency is less than 0.01% for an air gap of less than 2 microns. The graph shows the error increases to 1% with an air gap of 4.6 microns. For an air gap of 15 microns the error is 2%. The trajectory of the error curve stabilizes at 5% for air gaps bigger than 400 microns.

### 3.2. Enhancing the Sensitivity of the M-SRR Sensor

**Figure 9:**
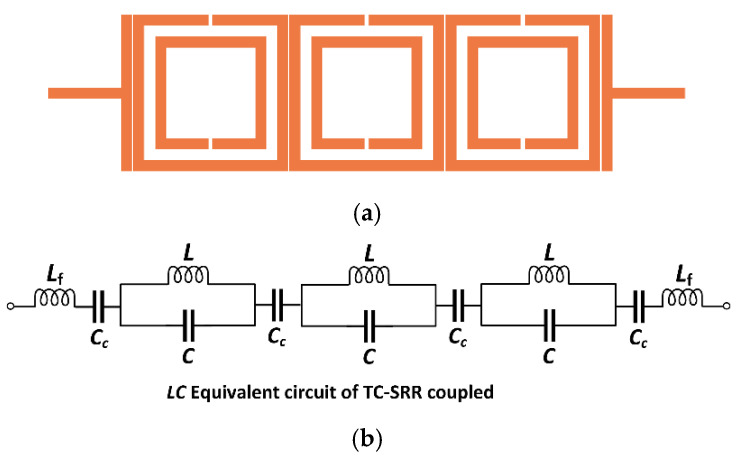
(**a**) Sensor based on the tri-composite split-ring resonator (TC-SRR). (**b**) Simplified equivalent circuit model of TC-SRR.

Material characterization at radio frequency (RF) signals has gained increasing importance in various fields including material science and biomedical research. Highly accurate sensors are needed for such applications. The performance of the M-SRR unit-cell sensor therefore needed to be improved to accurately measure the permittivity of the MUT. In this endeavor, the three unit-cells were cascaded together to create a tri-composite split-ring resonator (TC-SRR), as shown in Figure 9. Each of the split-ring resonators is essentially a folded half-wavelength resonator. Coupling in these structures is by proximity coupling through fringe fields. The nature and the strength of the fringe fields govern the magnitude and the strength of the coupling. It can be shown that at the resonance of the fundamental mode, the SRR has the maximum E-field density at the side with an open gap, and the maximum magnetic field density at the opposite side [25]. The fringe field diminishes rapidly away from the SRR. The fringe E-field is stronger near the side having the maximum E-field distribution, whereas the fringe H-field is stronger near the side having the maximum magnetic field distribution.

The total impedance (*Z_T_*) of the equivalent circuit model of the TC-SRR structure is given by:

(7) $$Z_{T}=j\left[\frac{3\omega L-4\left({1-\omega }^{2}L_{f}C_{c}\right)\left(1-\omega^{2}LC\right)}{\omega C_{c}(1-\omega^{2}LC)}\right]$$

The two resonance frequencies excited by TC-SRR are given by:

(8) $$f_{1}=\frac{1}{2\pi \sqrt{LC}}$$

(9) $$f_{2}=\frac{1}{2\pi \sqrt{L_{f}C_{c}}}$$

**Figure 10:**
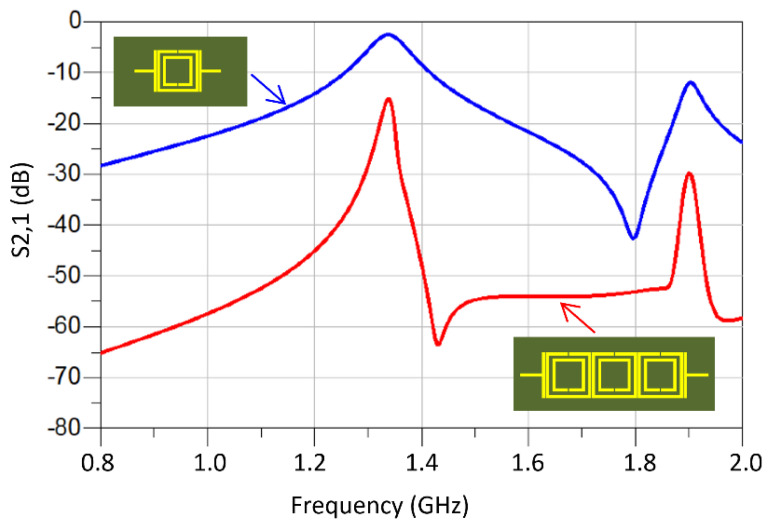
Transmission response of the M-SRR sensor and the TC-SRR sensor.

**Figure 11:**
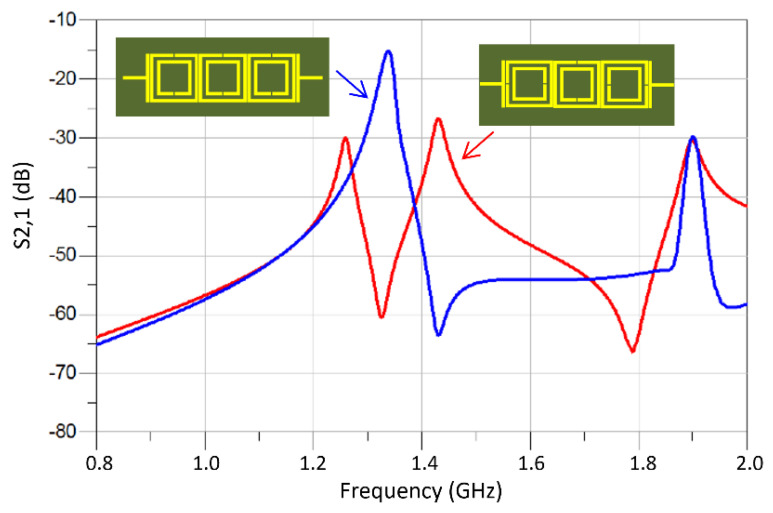
Transmission response of the TC-SRR sensor with no orthogonal unit-cell orientation and with orthogonal unit-cell orientation.

Compared in Figure 10 are the responses of the M-SRR unit-cell sensor and the TC-SRR sensor when constructed on an FR-4 substrate. It is clear that the TC-SRR sensor has a significantly sharper transmission response and is therefore a higher Q-factor than the M-SRR. Figure 11 shows what happens to the TC-SRR response when consecutive unit-cells are orthogonally oriented causing mixed coupling. Hence, the first mode splits into two resonance responses. The Q-factors of the split modes are marginally better than the unsplit mode; however, the modes have a much higher insertion-loss response.

### 3.3. Sensitivity Analysis of the TC-SRR Sensor

**Figure 12:**
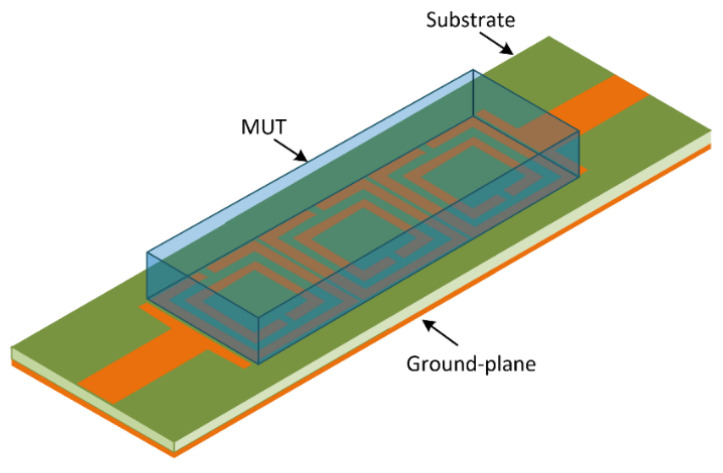
Setup of the TC-SRR sensor with MUT placed over the three SRR unit-cells.

**Figure 13:**
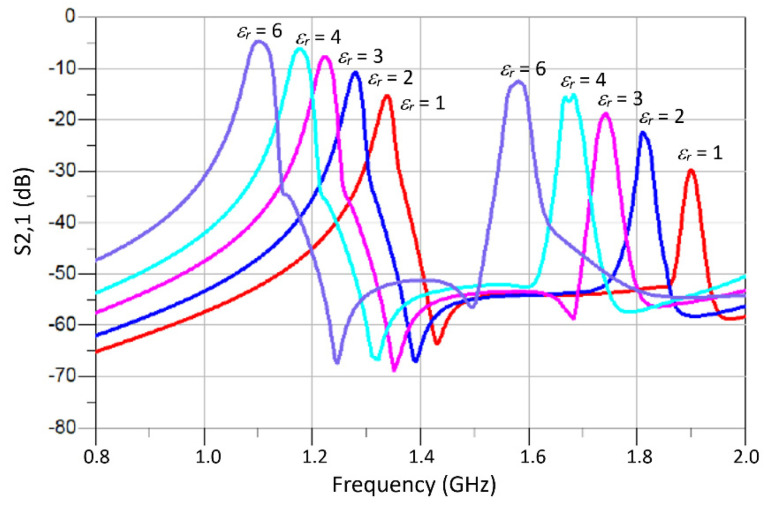
Simulated *S*_21_ for MUT samples with various permittivities and tan δ of 0.014.

**Figure 14:**
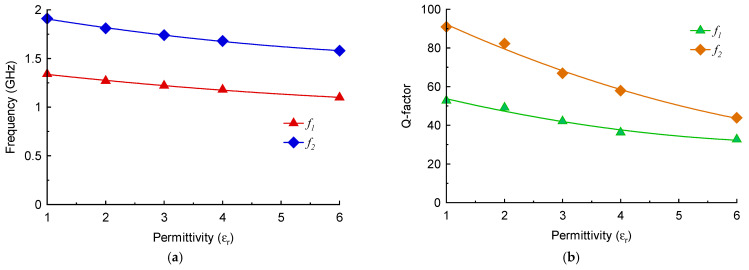
Sensitivity of the TC-SRR sensor. (**a**) Resonant frequencies as a function of permittivity, and (**b**) Q-factor as a function of permittivity.

**Figure 15:**
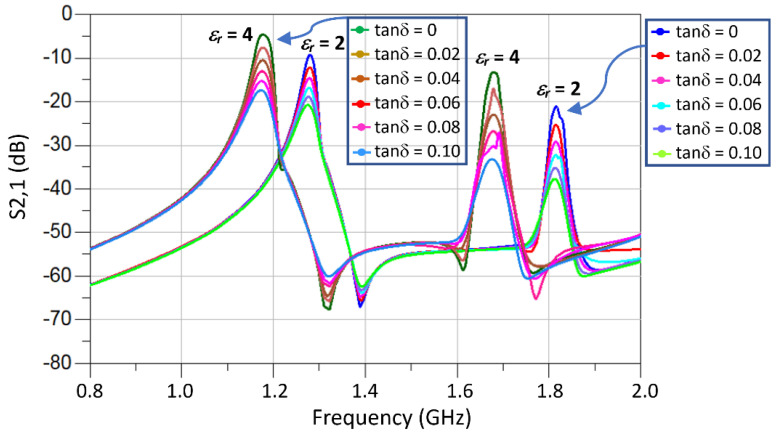
Insertion-loss response of the TC-SRR sensor for MUT samples of permittivities (*ε*_r_) 2 and 4 of various loss tangents.

**Figure 16:**
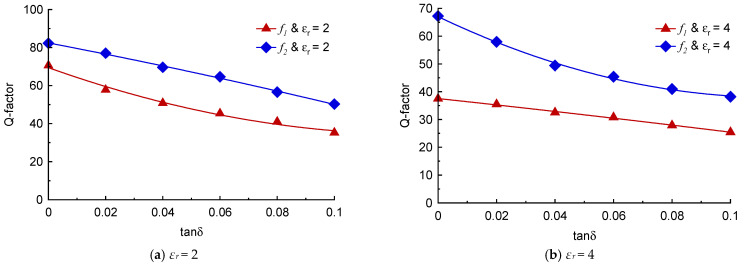
Q-factor versus loss tangent of TC-SRR sensor with MUT samples of permittivities (*ε_r_*) 2 and 4.

The material under test of a finite dimension, permittivity and loss tangent was loaded onto the TC-SRR sensor, as shown in Figure 12. The MUT covered the area under the three SRR unit-cells to perturb the sensor’s fringe EM-field and hence its resonance frequencies and insertion-loss. The change in the sensor’s properties enables the material characterization of the MUT. Figure 13 shows how the resonant frequencies of the TC-SRR sensor are affected by MUT samples with various material permittivities having a fixed tan δ of 0.014. The height of the MUT samples was fixed at 3 mm. Figure 13 shows that the increase in permittivity causes the resonance frequency and Q-factor to decrease and the insertion-loss to reduce. Figure 14a shows that the decrease in the resonance frequency of the first and second modes with increase in permittivity is almost linear; however, this is not the case for the Q-factor. The decrease in the Q-factor with the increase in the permittivity is much more pronounced for the second mode, as shown in Figure 14b. The effect on the loss-tangent on the sensor’s Q-factor and insertion-loss for MUT sample permittivities of 2 and 4 is shown in Figure 15. The increase in the loss-tangent results in higher insertion-loss; however, it has virtually no effect on the resonance frequency. The graph in Figure 16a shows that for a MUT sample of permittivity 2, the Q-factor of the second mode decreases more linearly with increases in the loss-tangent compared to the first mode. Figure 16b shows that for a higher permittivity of 4, the Q-factor decreases linearly with increases in the loss-tangent for the first mode; however, in the case of the second mode, the drop in the Q-factor follows an inverse relationship with an increase in the loss-tangent.

## 4. Measurements

**Figure 17:**
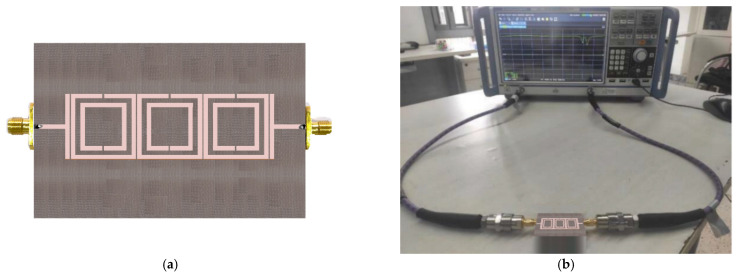
(**a**) Fabricated TC-SRR sensor. (**b**) Measurement setup for TC-SRR using R&S ZND Vector Network Analyzer.

**Figure 18:**
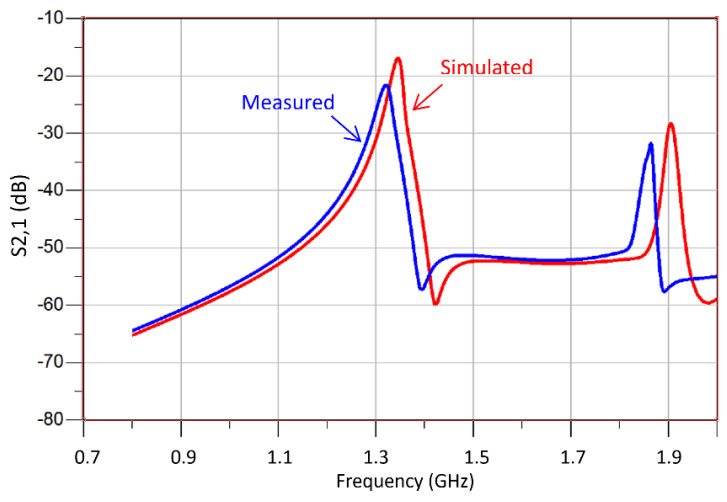
Measured and simulated transmission response (*S*_21_) of the TC-SRR sensor with no MUT sample.

The TC-SRR sensor was fabricated on an FR-4 substrate having a permittivity of 4.4, thickness of 1.6 mm and loss-tangent of 0.014 using the dimensions given in Table 1. Figure 17 shows the TC-SRR sensor connected for measurement to a Rohde and Schwarz ZND Vector Network Analyzer (VNA). The measured and simulated transmission responses are shown in Figure 18. The agreement between the measured and simulated results is very good. The discrepancy in the results is attributed to the manufacturing tolerances resulting in an error of less than 2% in the case of the first resonance frequency.

The accuracy of the proposed sensor in measuring the permittivity of various dielectric materials was assessed using the measurement setup shown in Figure 17b. The MUT samples selected for this test were commercial substrates, including RT/duroid 5870 [26] substrate [26], RT/duroid 5880 substrate [27], PTFE, Alumina etc. In the experiment the MUT substrates were of the size dimensions. The substrate samples were placed on top of the TC-SRR sensor and the shift in the resonance frequency of the first mode was measured. Using the data in Figure 14, the permittivity of the material was determined. Table 2 shows the measured and the manufacturer data. The error in the measurement of the permittivity is less than 1.5%. The accuracy of the proposed sensor is compared with recently reported planar sensors in Table 3. These results confirm that the measurement error of the proposed sensor is much smaller than other sensors, including our previous work [10]. Furthermore, the size of the sensor is comparable to other sensors. This sensor can be used to measure the permittivity of various chemicals and materials such as benzene (*ε_r_* = 2.3), acetic acid (*ε_r_* = 6.2), ethyl acetate (*ε_r_* = 6.4), polytetrafluoroethylene (*ε_r_* = 2), pine oil (*ε_r_* = 2.5), and sugar (*ε_r_* = 3).

## 5. Conclusions

The proposed sensor is based on a metamaterial structure consisting of a modified split-ring resonator (M-SRR). Sensitivity analysis of the M-SRR unit-cell was initially carriedout to characterize its behavior when it was subjected to sample materials of various permittivities. Because the sample material was placed on top of the sensor, there was a very small possibility of introducing a partial air gap that affects the accuracy of the sensor. As a result, it was necessary to analyze the effect of the air gap between the sensor and the sample material. The results reveal that an air gap of 2 microns can introduce an error of less than 0.01%. Under normal circumstances, there will be no air gaps between the sensor and the sample material. To enhance the sensitivity of the sensor, a tri-composite structure was developed based on the M-SRR unit-cell. The sensor was built and its performance was characterized. These data were used as a benchmark to determine the permittivities of sample materials. The sensor’s accuracy was tested with known dielectric materials. The error between the measured and published data was less than 2.5%, which is less than previously published works in the literature.

## Figures and Tables

**Table 1:** Dimensions of the proposed M-SRR unit-cell.

Parameters	Value (mm)
*d*	21.3
*W*	1.5
*g*	0.5
*c*	1.5
*s*	0.2

**Table 2:** Comparison between measured and manufacturer results.

Ref.	MUT	Permittivity (Real Part)		
		Measured	Published	Error (%)
[26]	Rogers RT/duroid 5870	2.30	2.33	1.29
[27]	Rogers RT/duroid 5880	2.17	2.2	1.36
[28]	PTFE	2.08	2.1	0.95
[29]	Alumina	9.78	9.9	1.21
[30]	Rogers RO3003	2.96	3	1.33
[31]	Rogers PR3006	6.07	6.15	1.3

**Table 3:** Comparison of worst-case error measurements with results from the literature.

Ref.	MUT	Freq. (GHz)	Size ${\left(\lambda_{o}\right)}^{2}$	Error in $\varepsilon_{r}$ Measurement (%)
[10]	Split-ring resonator	1.152	0.7 × 0.16	3.91
[32]	Split-ring resonator	1.2	0.48 × 0.16	2.91
[33]	Complementary square spiralresonator	2 & 5.41	0.45 × 0.54	5.0
[34]	Cascaded sensor array	4.84, 5.63, 6.97, 8.4	0.28 × 0.14	5.7
[35]	Split-ring	2.56	0.51 × 0.34	4.36
This work	Tri-composite split-ring resonator	1.32	0.32 × 0.12	1.36

## Data Availability

Not applicable.
